# Severe pulmonary insufficiency caused by Fallot-type absent pulmonary valve syndrome: A rare reason for neonatal central cyanosis

**DOI:** 10.1016/j.ijscr.2024.110076

**Published:** 2024-07-24

**Authors:** Mai Halloum, Saja Karaja, Ayham Qatza, Ahmed Aldolly, Aamer Razzouk, Saleh Takkem

**Affiliations:** a Faculty of Medicine, Hama University, Hama, Syria; b Department of Cardiology, Hama National Hospital, Ministry of health, Hama, Syria

**Keywords:** Cyanosis, Systolic ejection murmur, Absent pulmonary valve syndrome (APVS), Tetralogy of Fallot (TOF), Right ventricular hypertrophy (RVH), Ventricular septal defect (VSD), Case report

## Abstract

**Introduction:**

Absent Pulmonary Valve Syndrome (APVS) is a rare birth defect where the pulmonary valve is missing or underdeveloped. APVS often occurs alongside Tetralogy of Fallot, (TOF) another heart defect.

**Presentation of case:**

A 33-year-old woman gave birth to a male infant with severe pulmonary stenosis (PS) and a large ventricular septal defect (VSD). The infant underwent surgery to close the VSD and resect the stenotic ring. Two years later, he remained asymptomatic with a closed VSD and no pulmonary valve gradient.

**Discussion:**

Despite high mortality rates, long-term survival has improved with advancements in surgical repair. This case underscores the significance of early detection and personalized surgical strategies for complex congenital heart defects.

**Conclusion:**

Early identification of subtle symptoms is crucial for timely intervention, while individualized surgical strategies optimize outcomes. Further research is needed to understand the complex interplay of cardiac anomalies in APVS, particularly the absence of a patent ductus arteriosus in this case.

## Highlights

• The importance of observation and direct clinical survey of the newborn, there was a reason to detect a serious cardiac syndrome.
• Pulmonary artery dilatation over time turned dyspnea into severe pulmonary insufficiency.
• Cyanosis is a primary symptom in our patient, which was the result of severe respiratory insufficiency.
• The absence of a patent ductus arteriosus is not yet clearly explained for this syndrome.
• Interventional surgery is the gold standard, but individualizing each patient's case requires an individualized surgical approach and this is a major challenge for doctors.

## 1 Introduction

Absent pulmonary valve syndrome (APVS), first described by Cheevers in 1847, is a rare congenital heart disease [1]. It is characterized by the absence or underdevelopment of the pulmonary valve, yet it typically does not present with the usual cyanosis [2]. Without the pulmonary valve, blood moves inconsistently between the right ventricle and pulmonary arteries, causing the arteries to become abnormally large [3], which leads to a broad severity range, from severe newborn respiratory distress to mild wheezing in older children [2]. This syndrome Is commonly associated with an overarching congenital condition known as Tetralogy of Fallot (TOF), which involves multiple cardiac defects including a ventricular septal defect, right ventricular hypertrophy, and an overriding aorta [1]. Although overall mortality remains high, the rate of long-term survival has improved in recent years, leading to a decrease in deaths occurring after the first year following the surgical repair [4]. Here we present the case of an infant diagnosed with APVS and TOF who successfully underwent surgical repair and is now, two years later, In good health.

## 2 Presentation of case

**Fig. 1:**
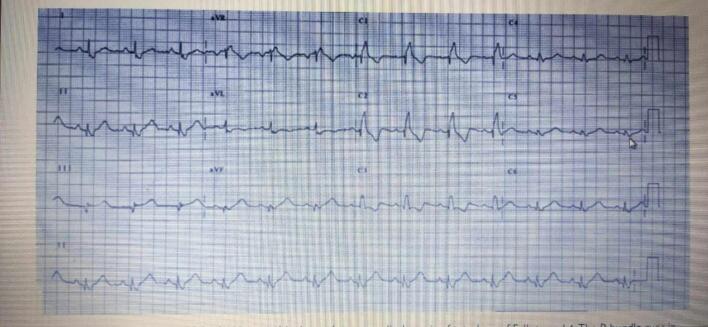
An ECG showing right ventricular hypertrophy (RVH) and right axis deviation (RAD).

**Fig. 2:**
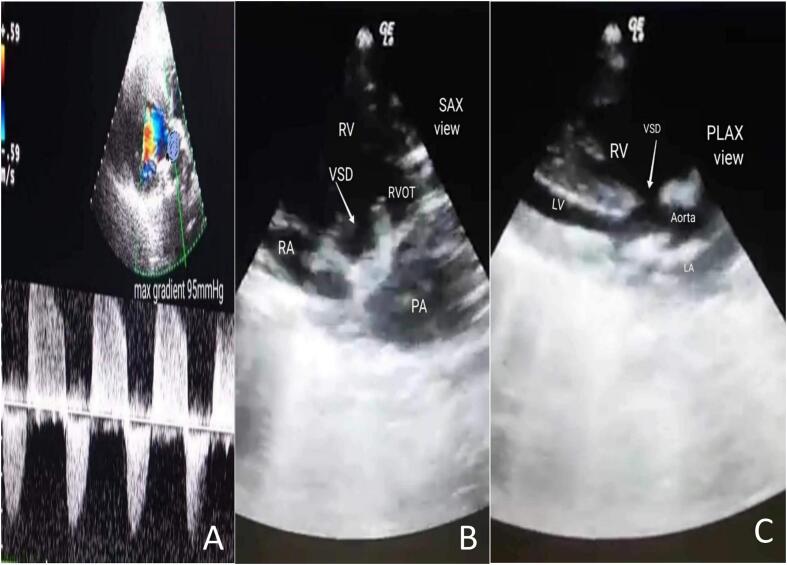

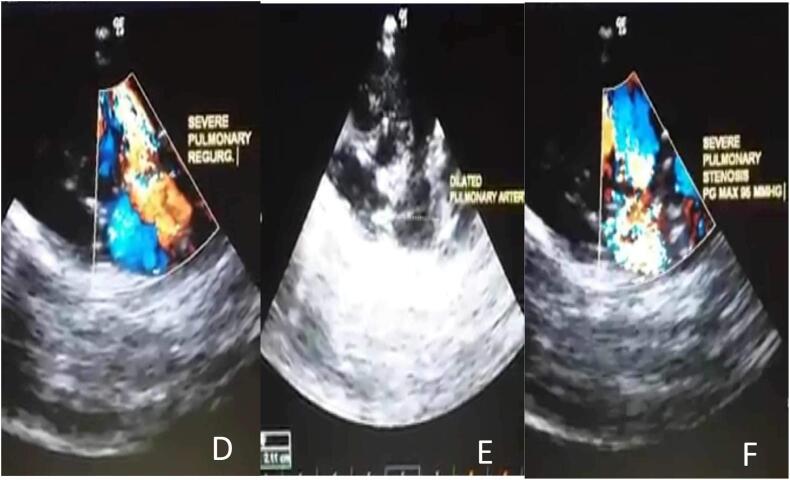

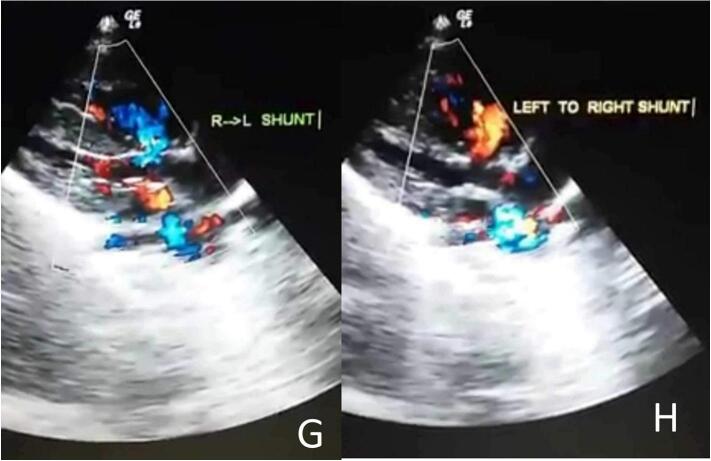
An echocardiogram Doppler image of a pulmonary valve showing severe pulmonary regurgitation and severe stenosis with a peak gradient of 95 mmHg (2-A). A parasternal short-axis view adjacent to the sternum, showing an Membranous ventricular septal defect (vsd), severe pulmonary arterial dilation, and a constricted ring in the area of the absent valve(2-B). The parasternal long-axis view, which is adjacent to the sternum, showed the type of defect along with malalignment of the outflow tract (2-C). The echocardiographic Doppler study demonstrates severe pulmonary regurgitation(2-D). The echocardiography shows a dilated pulmonary artery (2-E). The echocardiographic Doppler study demonstrates severe pulmonary stenosis with a maximum pressure gradient (PG max) of 95 mmHg (2-F). The parasternal long-axis in the color Doppler echocardiography reveals the presence of a right-to-left shunt (2-G). The color Doppler echocardiography's parasternal long-axis view reveals a significant defect causing a left-to-right shunt, characterized by a predominant flow from left to right (2-H).

A 33-year-old pregnant woman was born a male newborn by vaginal delivery at 38 weeks of gestation. On the third day, the neonate was referred to the cardiology department due to symptoms of central cyanosis, respiratory distress, and a left parasternal 3/6 systolic ejection murmur. She was three times pregnant, with no complications in either of the prior or current pregnancies. She underwent three previous vaginal deliveries. The medical, allergic, genetic, and psychosocial histories were within normal and she denied the use of tobacco, alcohol, and drugs. The neonate weighed 5000 g and his vital signs had been recorded: blood pressure, 70/55 mmHg; pulse, 110 beats/min; tympanic temperature, 37C; and oxygen saturation, 80 % on room air. Therefore, he was transferred to an incubator for 5 days to improve his general condition and oxygenation, which reached 85 %. Then he was referred for a full cardiac study. Laboratory blood and electrolyte tests were normal. A chest X-ray demonstrated an increased cardiothoracic ratio (CTR). Echocardiography (ECG) showed right axis deviation (RAD) and right ventricular hypertrophy (RVH) (Fig. 1). Echocardiography findings were as follows: superior vena cava (SVC) and inferior vena cava (IVC) flow into the right atrium and pulmonary veins flow into the left atrium with atrioventricular concordance and ventricular arterial concordance. Furthermore, the aorta, left aortic arch, left atrium and left ventricle, which measured 9 × 15 mm, were visible. Moderate dilatation of the right atrium and right ventricular (RV), which was accompanied by concentric hypertrophy, was observed. In addition, mild tricuspid valve insufficiency, absent ductus arteriosus, and absent pulmonary valve cusps had a severely stenotic fibrous ring In their place, which measured 9 mm in diameter(Fig. 2-B). Color and spectrum Doppler ultrasound showed that the peak flow velocity through the pulmonary valve annulus increased to 5 m/s and the gradient between the right ventricle and the PA was measured at 95 mmHg (Fig. 2-A, F). Severe dilatation was noted in the main pulmonary artery with a diameter of 21 mm and also in the dilatation of its branches, with the left pulmonary artery measuring 10 mm in diameter and the right pulmonary artery measuring 11 mm in diameter (Fig. 2-E). The ejection fraction (EF) was 70 %. There was a large membranous ventricular septal defect (VSD) of about 9.5 mm (Fig. 2-C, D) with a bi-directional shunt and a predominance of left to right during systole. The aorta overriding rate was about 50 % of the septal defect (Fig. 2-G, H) The patent foramen ovale (PFO) was 2 mm in diameter, and severe pulmonary insufficiency was noted. The neonate has no other abnormalities besides its heart. The diagnosis for the infant was thus APVS associated with TOF (TOF-type APVS) based on the following evidence: absent pulmonary valve, absent ductus arteriosus, and large membranous VSD. The procedure involved opening the right ventricle, addressing the stenotic pulmonary valve by widening the ventricular outlet with an artificial patch, and closing the interventricular opening with another patch and a stenotic fibrous ring was resected. It's important to note that the decision not to replace the pulmonary valve immediately is based on the consideration that the heart may change in size over time. The right ventricle may not tolerate stenosis but could tolerate insufficiency. Two years later, during his last follow-up, he was still asymptomatic from a cardiac standpoint and had no respiratory symptoms. An ECG was normal. The VSD was tightly closed without any leakage. In addition, there was an absence of the gradient across the pulmonary valve.

## 3 Discussion

Absent pulmonary valve syndrome (APVS) is a rare congenital heart disease (CHD) that presents with an absence of dysplastic pulmonary valve leaflets, aneurysmal pulmonary arteries (PA), and respiratory symptoms [5]. It occurs in 0.2–0.4 % of all live births with CHD [6], and in approximately 2.4 % to 6.3 % of cases with tetralogy of Fallot (TOF) [5]. APVS is commonly classified into two types. The first type, more common, is TOF-type APVS, which includes a ventricular septal defect (VSD), an overriding aorta, and the absence of ductus arteriosus (DA). The second type, less common, is non-TOF-type APVS which includes an intact ventricular septum, less severe PA dilation, and a patent DA, with or without tricuspid atresia. Herein, we discuss the patient's case with the first type of APVS (TOF-type APVS). The reason for the absence of DA remained uncertain [1]. During fetal development, the absence of DA blocks the flow of blood from the PA to the descending aorta. This leads to higher PA pressure and significant pulmonary regurgitation, impacting the normal development of the pulmonary valve [3]. According to the clinical courses and ages of patients, APVS is also categorized into two groups. Newborns in the first group suffer from dyspnea, recurrent lung infections, pulmonary emphysema, and atelectasis. The second group consists of older patients with mild symptoms who survived infancy. Later on, these patients may undergo minimally risky elective procedures to close their VSD and relieve their pulmonary stenosis [5]. Moreover, the cyanosis observed stems from both lung-related and heart-related diseases. Desaturation of blood in the pulmonary veins may be attributable to a mismatch in ventilation-perfusion and shunting within the lungs. Additionally, a right-to-left cardiac shunt at the ventricular level can also induce cyanosis, although it is uncommon for an obstruction in the right ventricular outflow tract (RVOT) to be significant enough to provoke a substantial right-to-left shunt [2]. Numerous newborns exhibit signs of respiratory distress immediately after birth. This condition is characterized by trapped air, causing the lungs to become overly inflated. As a result, ventilation becomes inadequate and the respiratory effort significantly increases [2]. In our case a membranous VSD of 9.5 mm (large) with malposition of the great vessels. Bidirectional shunt with left-to-right predominance. Despite the rarity of cyanosis, the patient exhibited central cyanosis along with respiratory distress. The primary pulmonary artery typically undergoes dilation, expanding to at least double or triple its normal width. Likewise, the right and left pulmonary artery branches, which usually measure between 4 and 5 mm in diameter, frequently exhibit an expansion to two or three times their standard size [2]. In our case, the diameter of the left pulmonary artery was 10 mm and the right was 11 mm.In cases of APVS, the area at the valve annulus is marked by the presence of rudimentary, myxomatous tissue remnants, rather than the actual development of proper valve leaflets [2]. In our case, the pulmonary Valve is absent at the level of the constricted fibrous ring with a diameter of 9 mm. An interesting note about APVS is that the DA is almost invariably absent in cases where the pulmonary arteries are connected In continuity [2], and This matches our case where there is also no patent ductus arteriosus present. The pulmonary artery and its branches may undergo aneurysmal dilation, which commonly exerts pressure on the bronchial tree and the esophagus, resulting in conditions such as bronchomalacia and polyhydramnios [2]. In our case, the internal organs were normal following the ultrasound examination. APVS is usually associated with a poor prognosis. According to one study, there is a strong correlation between respiratory complications and prognosis. Furthermore, the results of a different study indicated that the following complications were poor prognostic indicators of APVS: balloon-type pulmonary arterial morphology, hydrops fetalis, polyhydramnios, and bronchomalacia [1]. TOF-type APVS fetuses are typically diagnosed post-20 weeks of gestation through echocardiography, where color Doppler flow imaging (CDFI) reveals a distinct color mosaic pattern reflecting the “to-and-from” blood flow between the right ventricle and PA, except positive grayscale images. Fast blood flow, both antegrade and retrograde, is visible on a spectrum Doppler in PA [6]. Furthermore, in cases where other techniques, such as angiography, are impractical, it can serve as a preoperative assessment of APVS [1]. Chest X-ray findings may demonstrate lung hyperexpansion, with varying involvement of individual lobes or the entire lung based on the site of bronchial obstruction. Unilateral obstruction often results in a significant mediastinal shift towards the contralateral side. Computed tomography (CT) and magnetic resonance imaging (MRI) scans are useful to identify the exact relationship between central PA dilatation and airway compression locations. Bronchoscopy is a useful tool for assessing the extent of airway compression. Additionally, in cases of mild central PA dilation, it can help exclude tracheal and bronchial compression as potential causes of respiratory symptoms. The utilization of cardiac catheterization in APVS patients is rare [2]. In our case, chest X-rays revealed an increase in cardiothoracic ratio (CTR). In addition, echocardiography showed dilatation of left PA and right PA, absence of the pulmonary valve with a severely stenotic fibrous ring in its place, and retrograde blood flow from the PA trunk to RV. For children suffering from absent pulmonary valve syndrome, there is a lack of satisfactory palliative treatments [2]. When a newborn experiences significant respiratory distress immediately following birth, it necessitates either an emergency or a prompt surgical remedy. The optimal surgical approach is generally as outlined below. This involves a complete substitution of the main pulmonary arteries ron patch is applied using 5/0 Tevdek sutures with interrupted pledgeted horizontal mattress stitches, following standard Procedure [2]. It has been proposed that early surgical closure of the ductus arteriosus may enhance hemodynamic stability, thereby postponing the need for The monetary valve surgical procedure [1]. When an infant exhibits symptoms such as wheezing and recurrent respiratory infections, it's crucial to proceed with surgical intervention soon after confirming the diagnosis [2]. The outcomes of surgical treatment for absent pulmonary valve syndrome, a notably uncommon condition that appears across a broad range of severity, are challenging to predict due to the variability in risks associated with the surgical treatment of a particular child. The existence of various surgical methods developed during the early years of heart surgery indicates that the results were not optimal until the recent decade [2]. In our case, the VSD patch was closed and the fibrous valve ring was biopsied, while the pulmonary regurgitation was left untreated. After 10 years, if there Is severe right ventricular failure, a procedure to create a pulmonary valve from a living patch may be conducted. The child is currently two years old and in good condition post-surgery with the VSD securely closed without any leakage, no gradient across the pulmonary valve, and pulmonary regurgitation still present.

## 4 Conclusion

This case report demonstrates the successful surgical repair of a complex congenital heart defect in an infant with APVS with TOF. The case highlights the importance of meticulous observation and early detection of congenital heart conditions in newborns, as even subtle signs like cyanosis and dyspnea can indicate a serious cardiac syndrome. While surgical intervention is currently the gold standard, individualized surgical approaches are essential for optimizing outcomes, emphasizing the need for ongoing research and development of personalized treatment strategies for APVS with TOF patients. Further investigation into the absence of a patent ductus arteriosus in this case is warranted to better understand the complex interplay of cardiac anomalies in APVS.

## Abbreviations

APVS: Absent pulmonary valve syndrome
TOF: Tetralogy of Fallot
CTR: cardiothoracic ratio
ECG: Echocardiography
RAD: right axis deviation
RVH: Right ventricular hypertrophy
SVC: superior vena cava
IVC: inferior vena cava
RV: right ventricular
EF: ejection fraction
VSD: ventricular septal defect
PFO: patent foramen ovale
TOF-type APVS: APVS associated with TOF
CHD: congenital heart disease
PA: pulmonary arteries
DA: ductus arteriosus
RVOT: right ventricular outflow tract
CDFI: color Doppler flow imaging
CT: Computed tomography
MRI: magnetic resonance imaging

## Declaration of generative AI in scientific writing

None.

## Methods

The work has been reported in line with the SCARE criteria [7].

## Ethical approval

Not applicable.

## Funding

None.

## Author contribution

Mai Halloum: Data curation, Writing – review & editing, Writing – original draft.

Saja Karaja: Writing – review & editing, Writing – original draft.

Ayham Qatza: Writing – review & editing, Writing – original draft.

Ahmed Aldolly: Writing – review & editing, Writing – original draft.

Aamer Razzouk: Writing – review & editing, Writing – original draft.

Saleh Takkem: Writing – review & editing, Supervision.

All authors read and approved the final manuscript.

## Guarantor

Saja Karaja and Mai Halloum.

## Research registration number

Not applicable.

## Acknowledgements statement

None.

## Conflict of interest statement

None.
